# Single-stage transforaminal decompression, debridement, interbody fusion, and posterior instrumentation for lumbosacral brucellosis

**DOI:** 10.1186/s12893-017-0279-x

**Published:** 2017-07-14

**Authors:** Yakefu Abulizi, Wei-Dong Liang, Aikeremujiang Muheremu, Maierdan Maimaiti, Wei-Bin Sheng

**Affiliations:** 1 Department of spine surgery, First Affiliated Hospital of Xinjiang Medical University, Urumqi, Xinjiang Uyghur Autonomous Region 830054 China; 2 Department of spine surgery, Sixth’s Affiliated Hospital of Xinjiang Medical University, Urumqi, Xinjiang Uyghur Autonomous Region 830002 China

**Keywords:** Lumbosacral brucellosis, Transforaminal debridement, Interbody fusion, Posterior instrumentation

## Abstract

**Background:**

Spinal brucellosis is a less commonly reported infectious spinal pathology. There are few reports regarding the surgical treatment of spinal brucellosis in existing literature. This retrospective study was conducted to determine the effectiveness of single-stage transforaminal decompression, debridement, interbody fusion, and posterior instrumentation for lumbosacral spinal brucellosis.

**Methods:**

From February 2012 to April 2015, 32 consecutive patients (19 males and 13 females, mean age 53.7 ± 8.7) with lumbosacral brucellosis treated by transforaminal decompression, debridement, interbody fusion, and posterior instrumentation were enrolled. Medical records, imaging studies, laboratory data were collected and summarized. Surgical outcomes were evaluated based on visual analogue scale (VAS), Oswestry Disability Index (ODI) and Japanese Orthopaedic Association (JOA) scale. The changes in C-reactive protein (CRP) levels, erythrocyte sedimentation rate (ESR), clinical symptoms and complications were investigated. Graft fusion was evaluated using Bridwell grading criteria.

**Results:**

The mean follow-up period was 24.9 ± 8.2 months. Back pain and radiating leg pain was relieved significantly in all patients after operation. No implant failures were observed in any patients. Wound infection was observed in two patients and sinus formation was observed in one patient. Solid bony fusion was achieved in 30 patients and the fusion rate was 93.8%. The levels of ESR and CRP were returned to normal by the end of three months’ follow-up. VAS and ODI scores were significantly improved (*P* < 0.05). According to JOA score, surgical improvement was excellent in 22 cases (68.8%), good in 9 cases (28.1%), moderate in 1 case (3.1%) at the last follow-up.

**Conclusions:**

Single-stage transforaminal decompression, debridement, interbody fusion, and posterior instrumentation is an effective and safe approach for lumbosacral brucellosis.

## Background

Human brucellosis remains the most common zoonotic disease worldwide, with more than half a million new cases annually [1]. It is a systemic infection caused by facultative intracellular bacteria of the genus Brucella that can involve many organ systems. The disease mainly affects organs rich in reticuloendothelial cells, particularly the musculoskeletal system, which is the most frequent target site [2]. The incidence of musculoskeletal involvement of the genus Brucella varies significantly in the literature, ranging between 5 and 85% in most studies [3, 4]. Spinal brucellosis is one of the most frequent and severe manifestation of musculoskeletal involvement, which is also defined as the involvement of vertebral column, interspinal spaces, and/or paraspinal areas, and it occurs most commonly in the lumbosacral region [5].

The clinical features of spinal brucellosis are non-specific and can overlap with a wide spectrum of other infectious and non-infectious diseases [6]. Despite progress, timely and accurate diagnosis of spinal brucellosis continues to challenge clinicians because of its late-onset radiological findings, slow growth rate in blood cultures and the complexity of its serodiagnosis [7]. Therefore, spinal brucellosis remains under-diagnosed and under-reported. On the other hand, WHO has not updated its recommended treatment regimens for brucellosis in more than 20 years. Although some new therapeutic guidelines are recommended in literature, the optimal treatment regimen and duration of antibiotics remain controversial [8]. Frequent relapses, treatment failure, sequelae are reported constantly [9].

Although spinal brucellosis is rarely fatal, it can be severely debilitating and disabling. The disease can result in disc destruction, sclerosis of the vertebral body, and abscess formation [10]. If not treated appropriately, may cause serious sequelae such as chronic back pain, neurological deficit, and even kyphotic deformity [5]. Antibiotic therapy remains the mainstay for the treatment of spinal brucellosis, and usually has a good prognosis. However, cases with neurological dysfunction, spinal instability, abscess formation, intractable pain and failed response to conservative treatment may require surgical treatment. To our knowledge, surgical treatment of spinal brucellosis has rarely been reported. The purpose of this study was to determine clinical feasibility and effectiveness of single-stage transforaminal decompression, debridement, interbody fusion and posterior instrumentation for spinal brucellosis in lumbosacral region.

## Methods

### General information

A total number of 32 consecutive patients (19 males and 13 females, age: 53.7 ± 8.7, range 37-69 years) with lumbosacral brucellosis treated by single-stage transforaminal decompression, debridement, interbody fusion, and posterior instrumentation from February 2012 to April 2015 in our institution were included in this study (Table 1). Initial diagnosis of spinal brucellosis was based on the presence of findings consistent with infection in lumbosacral region on plain radiography, Computed tomography (CT) and Magnetic resonance imaging (MRI) (Table 2) and the confirmed diagnosis was made by isolation of Brucella species from blood and/or standard tube agglutination test and revealing a titer of antibodies to Brucella of ≥1/160.

**Table 1:** Basic information of all patients

Case no.	Age (years)	Gender	Duration of symptoms (months)	Level	Brucella agglutination test	Blood culture	Hospitalization (days)	Follow up (months)
1	59	M	3	L3-4	1/160	N	12	18
2	53	M	5	L4-5	1/160	N	10	24
3	56	M	8	L3-4	1/640	Y	10	12
4	50	F	6	L4-5	1/320	N	12	15
5	42	M	12	L4-5	1/320	N	9	36
6	62	M	12	L2-3	1/1280	Y	11	24
7	62	F	8	L4-5	1/160	Y	14	30
8	52	F	6	L5-S1	1/640	N	12	36
9	68	M	18	L2-3	1/640	N	15	18
10	49	M	8	L3-4	1/160	N	9	36
11	47	F	4	L4-5	1/320	N	11	24
12	49	M	8	L3-4	1/1280	N	11	18
13	47	M	12	L5-S1	1/640	N	13	24
14	48	F	6	L4-5,L5-S1	1/1280	Y	14	36
15	41	M	8	L3-4	1/320	N	10	38
16	51	F	24	L2-3	1/320	N	8	24
17	59	F	10	L5-S1	1/640	Y	13	12
18	42	M	6	L1-2,L4-5	1/320	N	13	36
19	69	F	3	L4-5	1/160	N	12	24
20	64	M	9	L2-4	1/640	N	14	18
21	53	M	6	L4-5	1/320	Y	9	30
22	69	M	18	L3-4	1/640	N	12	18
23	37	F	6	L5-S1	1/1280	N	11	36
24	58	F	12	L5-S1	1/640	N	14	18
25	39	M	9	L4-5	1/640	N	10	30
26	51	M	6	L5-S1	1/1280	Y	13	18
27	68	F	5	L4-5	1/640	N	9	12
28	55	F	8	L2-4	1/160	N	10	30
29	58	M	3	L5-S1	1/320	N	14	21
30	54	M	9	L5-S1	1/160	Y	11	36
31	52	F	10	L4-5	1/640	N	9	24
32	54	M	18	L1-2	1/160	N	14	21
Mean	53.7 ± 8.7		8.9 ± 4.9				11.5 ± 1.9	24.9 ± 8.2

*M* male, *F* female, *N* negative blood culture, *Y* positive blood culture

**Table 2:** Plain radiography, CT and MRI findings of all patients

Radiological studies	No. of patients (%)
X-ray	
-Narrowing of disc space	21(65.6%)
-End-plate lysis/sclerosis	9 (28.1%)
-Osteophyte formation	17 (53.1%)
-Destruction of vertebral body	6 (18.8%)
Computed tomography(CT)	
-Narrowing of disc space	21 (65.6%)
-End-plate lysis/sclerosis	19 (59.4%)
-Osteophyte formation	17 (53.1%)
-Destruction of vertebral body	15 (46.9%)
-Spinal canal stenosis	20 (62.5%)
-Sequestrum	4 (12.5%)
Magnetic resonance imaging(MRI)	
-Disc involvement	30 (93.8%)
-End-plate involvement	26 (81.3%)
-Destruction of vertebral body	28 (87.5%)
-Paravertebral abscess formation	7 (21.9%)
-Epidural granulation tissue or abscess	14 (43.8%)
-Spinal canal stenosis	23 (71.9%)

Indications for surgery in our study include: (1) persisting pain due to spinal instability which is caused by severe disc or/and vertebral destruction; (2) severe or progressive neurologic dysfunction; attributed to the nerve root compression by inflammatory granuloma, epidural abscesses; (3) the extensive or obvious paravertebral abscess formation or sequestrum formation in the vertebral body, no response to the antibiotic treatment; all cases were operated by the same senior author. Various parameters including blood loss, surgical time and intraoperative complications were recorded.

### Operative procedure

**Fig. 1:**
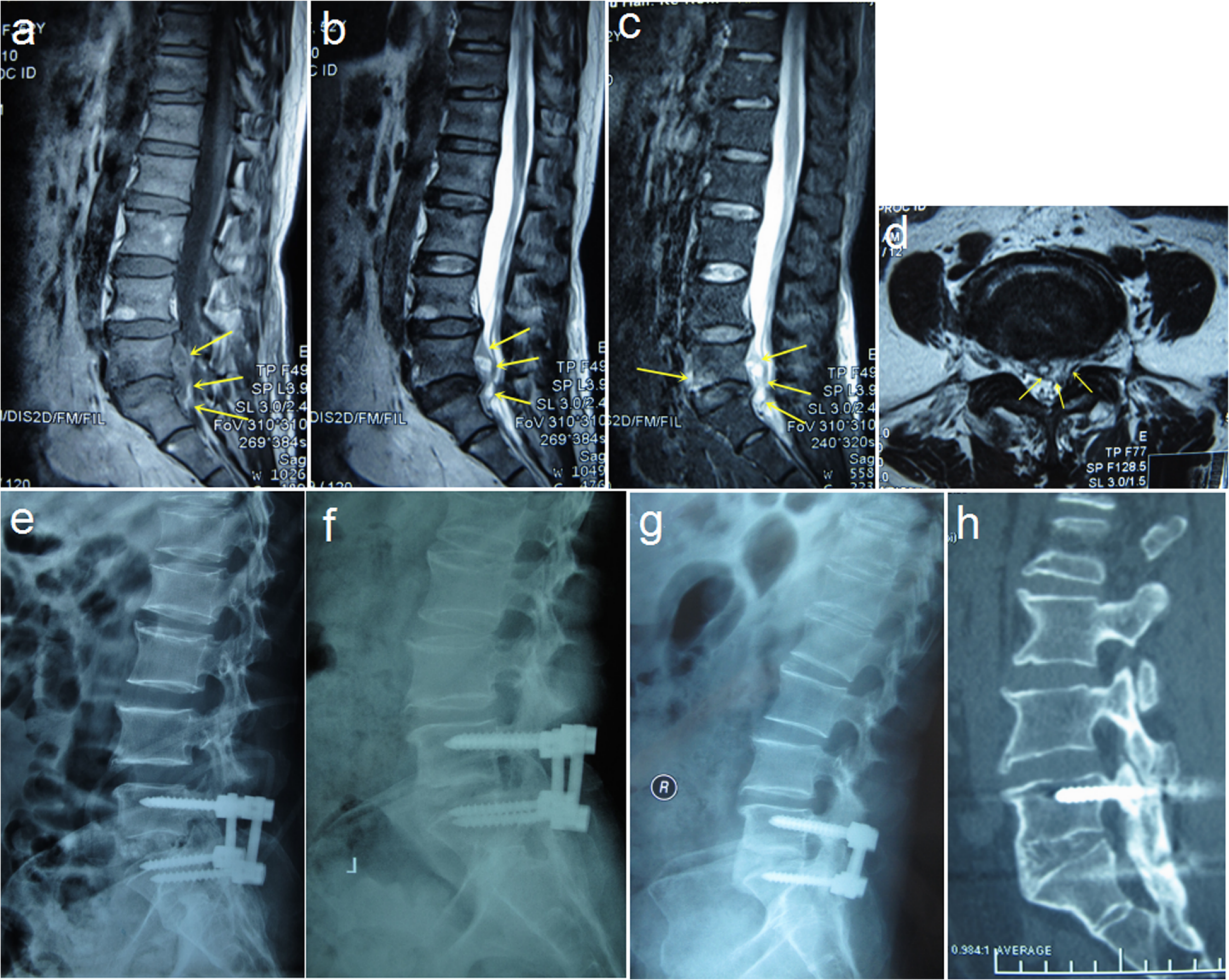
A 52-year-old female misdiagnosed as “disc herniation” at the first visit. After 4-month conservative treatment, the clinical symptoms aggravated. **a**, **b**, **c** T1,T2-weighted and STIR MRI showed inflammation in L5–S1 vertebral bodies and intervertebral disc. Spinal epidural abscess and inflammatory granuloma extend to anterior epidural space, resulting in spinal stenosis at L5-S1 level; (**d**) Transverse T2-weighted MRI showed left nerve root and dural sac compression; (**e**) postoperative X-ray showed intervertebral bone grafting and instrumentation; (**f**, **g**) 6-month and 36-month postoperative X-ray showed solid bony fusion. (**h**) the last follow-up CT showed solid intervertebral and facet joint bony fusion

**Fig.2:**
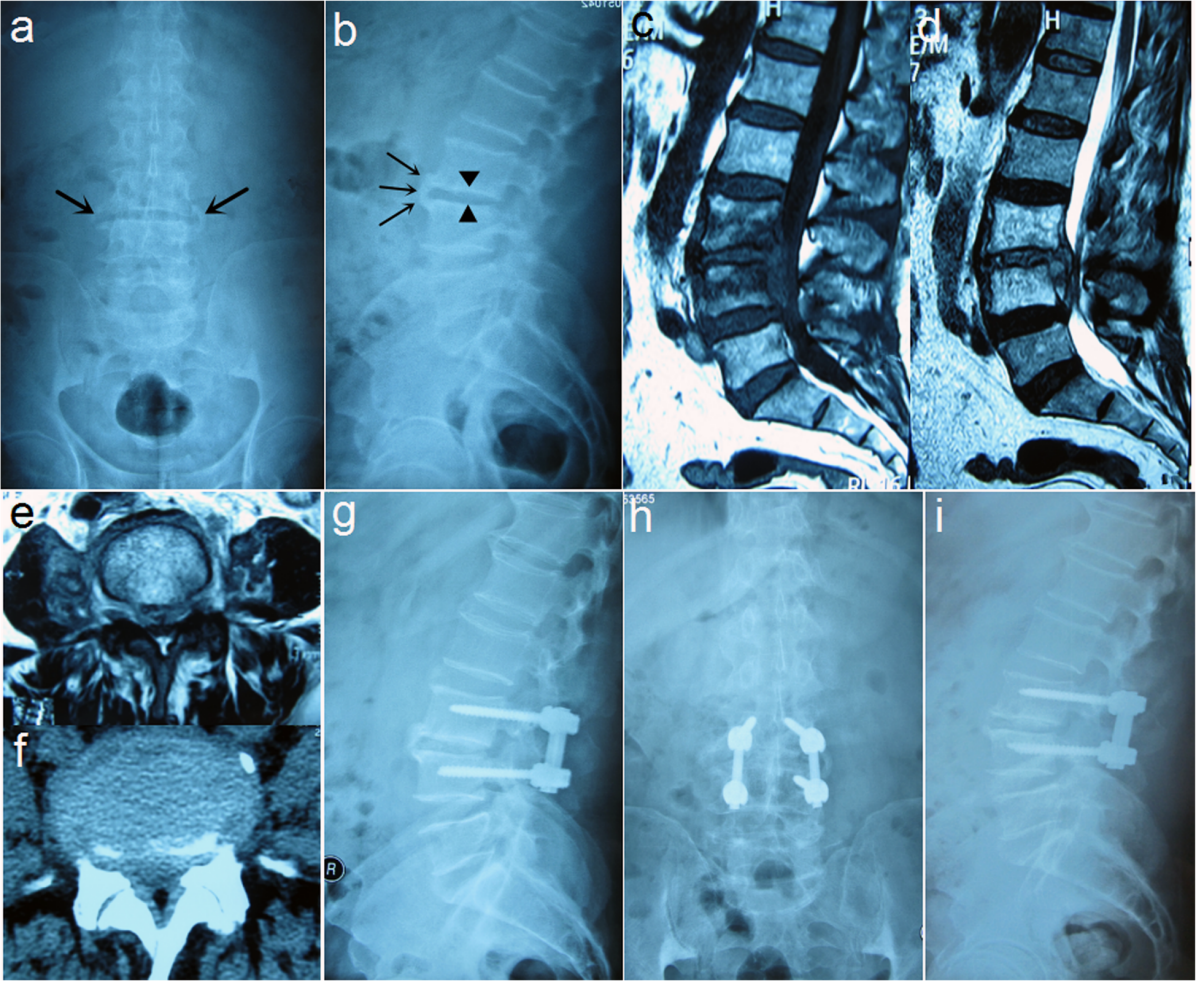
A 69-year-old male present with L3-L4 spinal brucellosis; (**a**) Anteroposterior view showed hyperplastic changes occur on the lateral edge of L3-L4 vertebral body, leading to the formation of osteophytes (*arrow*); (**b**) Lateral view showed disc space narrowing and anterior osteophyte formation (parrot’s beak); (**c**, **d**) Sagittal T1 and T2 weighted MRI images showed lesions involving in L3-L4 vertebral bodies and intervertebral disc. Epidural abscess and inflammatory granuloma formation; (**e**, **f**) Transverse MRI and CT images demonstrated spinal canal stenosis; (**g**, **h**) Postoperative X-ray showed intervertebral bone grafting and instrumentation; (**i**) 12-month postoperative X-ray showed a good fixed position and interbody fusion

All the surgeries were carried out under general anesthesia with endotracheal intubation. Each patient was placed on a four-poster spinal frame in the prone position. A midline longitudinal incision was made over the spinous process of the infected vertebra. The posterior spinal construction including the spinous processes, lamina, and facet joints was exposed and the transverse processes were left intact. Pedicle screws were placed into both sides of the affected vertebra with the assistance of c-arm fluoroscopy. In order to achieve adequate debridement, the pedicle screws were placed as close to the superior or inferior end plate as possible to keep them away from the site of infection. The screws were fixed to a temporary rod on one side where neurologic and radiological manifestation was less severe. A facetectomy was then performed at the involved level on the other side where bony destruction, epidural abscess, and radiculopathy were severe (Fig. 1a-d). The epidural abscess, infected disc, end plates, sequestrum and granulation tissues were debrided and the psoas abscess was drained posterolaterally under blunt dissection as thoroughly as possible. Removed tissues and abscess were sent for histopathologic examination. If the debridement was not sufficient under a unilateral facetectomy, or compression of both side nerve roots at the involved level was equally serious (Fig. 2c-f), the same debridement and decompression procedure was performed on the opposite side. After adequate removal of the lesion, the upper and lower bony recipient site were prepared by curettage until pinpoint bleeding. Then autologous bone or allograft was implanted in the defected space for interbody fusion. Pedicle screws on both sides were fixed to precontoured rods under compression. Local antibiotic therapy with 0.75-1.5 g streptomycin was routinely used in the surgical area. Drainage and incision sutures were performed postoperatively.

### Postoperative management

Intravenous antibiotics were routinely administered for one to three days postoperatively. The drainage tube was usually removed when volume was less than 30 ml/ day. All patients received the WHO recommended oral regimen, consisting of 200 mg doxycycline plus 600-900 mg rifampicin, for a minimum 3 months after operation. All patients remained in bed for 3-5 days and then mobilized under the effective support of a lumbosacral brace. X-ray examination was performed in all patients before discharge to evaluate the location of the graft and instrumentation (Fig. 1e, Fig. 2g-h). Brace protection continued for 2-3 months.

### Follow-up evaluation and statistical analysis

Patients were followed-up at 1, 3, 6 months and then annually. Medical records, imaging studies, laboratory data, neurologic and functional data were recorded and analyzed. The activity of infection was monitored with erythrocyte sedimentation rates (ESRs), and C-reactive protein (CRP) .The interbody fusion was evaluated by radiographs at the last follow-up (Fig. 1f-g, Fig. 2i), and the radiologic criteria of Bridwell et al. was used to assess graft fusion. When there was uncertainty on X-ray observation, CT scans was performed (Fig. 1h). The visual analogue scale (VAS) was used to assess the back pain. Pain-related dysfunction was assessed using the Oswestry Disability Index (ODI). Japanese Orthopaedic Association (JOA) scale was used to evaluate the functional outcomes.

All statistical analyses were performed with SPSS 20.0 statistical software. The Student’s *t* test was used to evaluate preoperative and final follow-up changes in laboratory (ESR, CRP) and quantitative scores (VAS, ODI, JOA). Any discrepancy in normal distribution was analyzed using the rank sum test. Statistical significance was set at *P* < 0.05.

## Results

Brucella agglutination test was ≥1/160 in all cases and blood culture was positive in 8 cases (25%). The clinical symptoms are summarized in (Table 3). Surgery was successfully carried out in all patients. The mean duration of surgery was 133.1 ± 36.6 min, and the mean blood loss was 378.1 ± 187.9 ml (range 120-800 ml). All histopathologic examinations revealed noncaseating granulomatous inflammation. All patients were available for follow-up for at least 12 months, mean 24.9 ± 8.2 months. Superficial wound infection, which may caused by the poor general condition of the patient, was observed in one patient at ten days after surgery and was successfully treated by intravenous antibiotic treatment. One patient had deep wound infections at 2 months after discharge and one patient had sinus formation at 3 months after the surgery. Both of these patients had a history of diabetes, and were cured by revision surgery and extended intravenous antibiotic infusion. No clinical or radiological relapses were found during the follow-up period.

**Table 3:** Clinical features of analyzed patients

Symptoms	No. of patients (%)
Spinal symptoms	
-Back pain	31 (96.9%)
-Radiculopathy	22 (68.8%)
constitutional symptoms	
-fever	27 (84.4%)
-Sweating	18 (56.3%)
-Weakness or fatigue	14 (43.8%)
-Weight loss	9 (28.1%)
-Hepatomegaly	7 (21.9%)
-Arthralgia	4 (12.5%)

The radiation pain of lower extremities was relieved on the same day after surgery. All patients had significant improvement in constitutional symptoms and back pain at the first month follow-up visit. ESR and CRP returned to normal levels in all patients within 3 months after surgery. The preoperative levels of ESR, CRP were 46.03 ± 12.73, 41.47 ± 41.74, and respectively declined to 8.86 ± 3.05, 4.56 ± 1.75 at the third month follow-up. Statistical analysis demonstrated that there were significant differences between preoperative and final follow-up VAS, ODI, and JOA scores (*P* < 0.05) (Table 4).

**Table 4:** Comparison of preoperative and last follow up VAS, ODI, JOA scores

Parameters	Preoperative	Last follow up	Improvement rate (%)	*P* value
VAS	5.19 ± 1.47	0.47 ± 0.67	90.9	<0.05
ODI	55.31 ± 9.16	10.72 ± 3.23	80.7	<0.05
JOA	12.38 ± 2.98	26.13 ± 2.58	82.7	<0.05

Scores were demonstrated as Mean ± Standard deviation

*VAS* visual analogue scale, *ODI* Oswestry Disability Index, *JOA* Japanese Orthopaedic Association

The interbody fusion status was evaluated at the last follow-up. According to Bridwell criteria, the degree of fusion was grade I in 30 patients and grade II in the remaining 2 patients. The total fusion rate was 93.8%. Lateral flexion/extension radiography and CT examinations were performed for these 2 patients, and did not find any detectable movement or gap in the interbody area.

## Discussion

Spinal brucellosis is one of the most common presentations of human brucellosis, whose incidence has been increasing rapidly around the world over the last decade, especially in the underdeveloped regions [11]. In spite of its high prevalence, and the availability of effective antibiotics for brucellosis, diagnosis and treatment of the disease still poses great challenge for the clinicians [12]. Anatomically, inflammation in spinal brucellosis first occurs at the anterior superior endplate, an area morphologically rich in blood supply, and extends to the entire vertebrae, adjacent disc/vertebrae and epidural space. The pathological process is very similar to tuberculostic infection, which is the “great imitator” of this disease. However, spinal brucellosis is relatively less destructive than spinal tuberculosis, and usually has good response to the antibiotic treatment [13]. Generally, most of the patients with spinal brucellosis can be treated nonoperatively. Koubaa M et al. reported 32 cases with spinal brucellosis that were treated by antibiotic treatment alone. In their a mean of 30 months’ follow-up, resolution of epidural or paravertebral masses was achieved in all cases without any clinical or radiological relapses [14].

However, because of delayed diagnosis and treatment, some cases with spinal brucellosis may develop with neurological deficit, persistent or progressive back pain due to spinal instability, and large paravertebral abscesses formation. In addition, there are a few cases that present with non-responsiveness to antibiotic treatment. For such patients, surgical treatment should be considered as the last resort [15, 16]. Nevertheless, surgical treatment of spinal brucellosis has been rarely reported in the literature, and the role of surgical intervention still remains controversial. In the current study, the surgery was performed with the aim of debriding the focus of infection entirely, recovering nerve function, reconstructing spinal stability and restoring normal sagittal alignment.

Spinal brucellosis typically occurs in the lumbosacral region, particularly at the L4–L5 and L5-S1 levels [3–5]. In our study, involvement of L4–L5 and L5-S1 levels account for 34% and 28% all cases respectively. Various methods of surgical debridement and fusion have been described for the infectious disease of lumbar spine including anterior, posterior and combined approaches. However, most of the previous studies focused on spinal tuberculosis. Anterior approach is considered the gold standard for debridement and decompression in infectious diseases. It allows direct access to the pathology, adequate debridement, adequate decompression, and the ability to place a large graft. But it is more challenging in patients with lumbarsacral spinal infections due to the complicated regional anatomy, and may increase the risk of surgery-related morbidity [17]. Also, anterior instrumentation at L4-L5 and L5-S1 is potentially dangerous and insubstantial [18]. In addition, most of the patients in our study showed bilateral nerve root compression, destructed disc herniation, epidural inflammatory granuloma or abscess formation, resulting in spinal stenosis. Anterior approach may not be adequate to achieve complete decompression of the contralateral nerve root. Furthermore, owing to the less destructive characteristics of spinal brucellosis, comparing with other infectious spinal disease, surgical intervention can be much easier and minimally invasive.

Posterior-only approaches are increasingly reported as an alternative method for selective spinal tuberculosis. Lee et al. have reported satisfactory results of traditional posterior lumbar interbody fusion (PLIF) and posterior instrumentation for lumbar spine tuberculosis [19]. TLIF has been developed as a modification of PLIF and has several advantages including minimizing traction on the nerve root and dura, lower rate of neurological injury, and lower risk of complications [20]. Currently, single-stage transforaminal decompression, debridement, interbody fusion, and posterior instrumentation is widely used in thoracic and lumbar spine tuberculosis, but it has been blamed to be inadequate to achieve satisfactory debridement and reconstruction of anterior column defects, and considered not suitable for some cases with large paravertebral abscess and severe vertebral collapse [21, 22]. However, with further studies with the characteristics of spinal brucellosis, we were convinced that TLIF is applicable for lumbarsacral brucellosis. In our series, no intraoperative complications were observed, and back pain and radiating leg pain were relieved significantly in all patients after surgery. The improvement rates of VAS, ODI scores were 90.9%, 80.7% respectively. Furthermore, no patient experienced recurrence of the disease during the follow-up.

The lesion of spinal brucellosis mainly located in the intervertebral space. Therefore, the operating space obtained by resection of both sides of the facet joint is sufficient for thorough removal of the pathology and complete decompression of bilateral nerve roots. Also, graft bone implantation can be easily accomplished via the path that runs through the far-lateral portion of the vertebral foramen. Moreover, in most cases of spinal brucellosis, destruction of vertebral body is not severe and often limited in the endplates of involved segments. Thus, transpedicular screws can be placed in the affected vertebra, which would allow minimal surgical exposure and short segmental fixation, and can save motion segments. As we know, interbody fusion and posterior instrumentation can provide reliable segmental stability and acceptable interbody fusion rates. In our study, the average time for bed rest was only 3.78 ± 1.68 days, and all patients were able to walk before discharge. At the last follow-up, 30 patients achieved solid fusion, and the total fusion rate was 93.8%. All patients returned to normal activities. According to JOA score, surgical improvement was excellent in 22 cases (68.8%), good in 9 cases (28.1%) and the improvement rate was 82.7%.

## Conclusion

For the patients with definitive surgical indications of lumbosacral brucellosis, single-stage transforaminal decompression, debridement, interbody fusion, and posterior instrumentation is an effective and safe surgical technique that should be considered as a choice for the treatment of lumbosacral brucellosis.

## Abbreviations

CRP: C-reactive protein
CT: Computed tomography
ESR: Erythrocyte sedimentation rate
JOA: Japanese orthopaedic association
MRI: Magnetic resonance imaging
ODI: Oswestry disability index
VAS: Visual analogue scale
WHO: World Health Organization
